# Comparative analysis of delivered and planned doses in target volumes for lung stereotactic ablative radiotherapy

**DOI:** 10.1186/s13014-024-02505-7

**Published:** 2024-08-16

**Authors:** Geum Bong Yu, Jung In Kim, Hak Jae Kim, Seungwan Lee, Chang Heon Choi, Seonghee Kang

**Affiliations:** 1 Department of Radiation Oncology, Seoul National University Hospital, 101, Daehak-ro, Jongno- gu, Seoul, 03080 Republic of Korea; 2 Institute of Radiation Medicine, Seoul National University Medical Research Center, Seoul, 03080 South Korea; 3 Biomedical Research Institute, Seoul National University Hospital, Seoul, 03080 South Korea; 4 Department of Radiation Oncology, Seoul National University College of Medicine, Seoul, 03080 Korea; 5 Cancer Research Institute, Seoul National University College of Medicine, Seoul, 03080 Korea; 6 Department of Radiological Science, Konyang University, Nonsan, 35365 South Korea

**Keywords:** Deformable image registration, Lung SABR, Dose evaluation, Adaptive CT

## Abstract

**Background:**

Adaptive therapy has been enormously improved based on the art of generating adaptive computed tomography (ACT) from planning CT (PCT) and the on-board image used for the patient setup. Exploiting the ACT, this study evaluated the dose delivered to patients with non-small-cell lung cancer (NSCLC) patients treated with stereotactic ablative radiotherapy (SABR) and derived relationship between the delivered dose and the parameters obtained through the evaluation procedure.

**Methods:**

SABR treatment records of 72 patients with NSCLC who were prescribed a dose of 60 Gy (D_prescribed_) to the 95% volume of the planning target volume (PTV) in four fractions were analysed in this retrospective study; 288 ACTs were generated by rigid and deformable registration of a PCT to a cone-beam computed tomography (CBCT) per fraction. Each ACT was sent to the treatment planning system (TPS) and treated as an individual PCT to calculate the dose. Delivered dose to a patient was estimated by averaging four doses calculated from four ACTs per treatment. Through the process, each ACT provided the geometric parameters, such as mean displacement of the deformed PTV voxels (Warp_mean_) and Dice similarity coefficient (DSC) from deformation vector field, and dosimetric parameters, e.g. difference of homogeneity index (ΔHI, HI defined as (D_2%_-D_98%_)/D_prescribed_*100) and mean delivered dose to the PTV (D_mean_), obtained from the dose statistics in the TPS. Those parameters were analyzed using multiple linear regression and one-way-ANOVA of SPSS^®^ (version 27).

**Results:**

The prescribed dose was confirmed to be fully delivered to internal target volume (ITV) within maximum difference of 1%, and the difference between the planned and delivered doses to the PTV was agreed within 6% for more than 95% of the ACT cases. Volume changes of the ITV during the treatment course were observed to be minor in comparison of their standard deviations. Multiple linear regression analysis between the obtained parameters and the dose delivered to 95% volume of the PTV (D_95%_) revealed four PTV parameters [Warp_mean_, DSC, ΔHI between the PCT and ACT, D_mean_] and the PTV D_95%_ to be significantly related with P-values < 0.05. The ACT cases of high ΔHI were caused by higher values of the Warp_mean_ and DSC from the deformable image registration, resulting in lower PTV D_95%_ delivered. The mean values of PTV D_95%_ and Warp_mean_ showed significant differences depending on the lung lobe where the tumour was located.

**Conclusions:**

Evaluation of the dose delivered to patients with NSCLC treated with SABR using ACTs confirmed that the prescribed dose was accurately delivered to the ITV. However, for the PTV, certain ACT cases characterised by high HI deviations from the original plan demonstrated variations in the delivered dose. These variations may potentially arise from factors such as patient setup during treatment, as suggested by the statistical analyses of the parameters obtained from the dose evaluation process.

## Background

### Stereotactic ablative radiotherapy treatment for patients with non-small cell lung cancer

Stereotactic ablative radiotherapy (SABR), also known as stereotactic body radiation therapy (SBRT), is a radiotherapy procedure that is highly effective in controlling early stage primary or oligometastatic cancers. It delivers a higher biologically effective dose to the tumour than does conventional radiotherapy [1–3]. Advancements in technologies, such as intensity-modulated radiation therapy (IMRT) and image-guided radiation therapy (IGRT) have helped achieve treatment goals by maintaining an acceptable therapeutic ratio with excellent dose conformity in SABR. An accurate patient setup facilitated by IGRT is a crucial aspect of SABR. Owing to advancements in IGRT, SABR has been applied to patients with early stage non-small cell lung cancer (NSCLC) who are medically inoperable or prefer non-invasive treatment [4–8].

### Adaptive radiation therapy with CBCT

One of the most critical aspects of IGRT is the application of imaging techniques for precise patient positioning during treatment sessions. In external beam radiation therapy, cone-beam computed tomography (CBCT), which is integrated with a linear accelerator, is extensively used to position a patient based on the stance taken during treatment simulation. The CBCT image can provide information on the patient’s treatment position, allowing estimation of the dose delivered to the gross tumour volume (GTV) and surrounding organs at risk (OARs). However, because of the known limitations of CBCT, such as the large uncertainty of the CT numbers arising from scattered photons [9–11] and several other effects [12–14], CBCT is considered inappropriate for direct dose computation [15]. This limitation prevents CBCT from being used as a planned CT for adaptive radiation therapy when the patient’s anatomy has been seriously deformed throughout the treatment course.

Several studies have attempted to overcome the limitations of CBCT. Things RS et al. [16] summarised various methods for utilising CBCTs for dose calculation, including patient-specific CBCT calibration [15, 17–20], bulk density override of tissues in the CBCT image [20], physics-based artefact corrections [21], histogram matching [22] and deep learning methods [23–25], and deformable image registration (DIR) of planning CT (PCT) to the daily CBCT [26–28].

Advances in the DIR algorithm and CBCT image quality [29–32] have enabled the accumulation of the delivered dose using the daily CBCT [33, 34] in addition to the efforts to overcome the limitations of CBCT. Based on the delivered dose estimation to the GTV and OARs, adaptive radiation therapy facilitates the modification of treatment plans to achieve optimal outcomes by adapting to the observed anatomic or physiologic variations in patients from the initial simulation.

### Delivered dose estimation using deformable image registration

Synthetic CT, also called adaptive CT (ACT), can be acquired by aligning and deforming the PCT onto daily CBCT. This process involves both rigid and deformable image registrations. In SABR, CBCT acquired for each patient’s setup at every fraction enables the creation of the ACT, allowing for the calculation of daily doses that reflect any variations from the initial simulation, and ultimately, the prediction of the total dose actually delivered.

Previous studies [35, 36] have evaluated the dose delivered to patients with NSCLC using the same commercial software. They either focused on a limited number of PTV parameters or examined only a few treatment sessions. This study aimed to assess the dose delivered throughout the treatment course, reflecting the patient’s anatomic and physiologic variations. Furthermore, maximum possible PTV parameters were collated through the dose evaluation process and were analysed to understand the relationship between the dose delivered to the PTV in each fraction and various parameters. This approach can help to identify the factors contributing to the observed dose distribution.

## Methods

### Collecting patient records

After obtaining the institutional review board approval, the treatment records of 72 patients with NSCLC were collected. The patients were treated with 60 Gy (D_prescribed_) in four fractions between 2019 and 2023, using volumetric arc-modulated therapy of a 6 MV flattening filter-free photon beam of Varian TrueBeam^STX^ (Varian Medical Systems Inc., Palo Alto, CA, USA). The D_prescribed_ was prescribed to the 95% volume of the PTV. For lung SABR treatment planning, the four-dimensional CTs (4DCTs) obtained with thoracic scan protocol were reconstructed into 10 respiratory phases by Brilliance CT Big Bore™ (Royal Philips Electronics, Amsterdam, Netherlands), and then their average CT image was generated for the treatment plan. The maximum intensity projection (MIP) of the tumour from each phase was integrated and contoured as an ITV, and the PTV was determined by embracing the ITV on a patient-by-patient basis with a 5–7-mm margin, compensating for several treatment uncertainties. Treatment plans were created using a treatment planning system (TPS, Eclipse Ver. 13.5 and 16.1 with Acuros XB algorithm, Varian Medical Systems Inc., Palo Alto, CA, USA). In the treatment room, the patients were positioned on the couch by matching the acquired daily CBCT image to the PCT image before treatment. The specifications of the PCT and CBCT images are listed in Table 1. The treatment records, including a set of Dicom RT plan (RP), RT dose (RD), RT structures (RS), the PCT, and daily CBCT images were exported into the Velocity software (Ver. 4.1, Varian Medical Systems Inc., Palo Alto, CA, USA) to generate ACT.

**Table 1:** Specifications of the treatment simulation CT and cone beam CT

In machine	Planning CT (PCT)	Cone beam CT
Manufacturer	Philips Electronics (Amsterdam, the Netherlands)	Varian Medical Systems (Palo Alto, CA, USA)
Model (software version)	Brilliance CT Big Bore™ (Ver. 4.2, 4.8)	TrueBeam^STX^ On Board Imager (Ver. 2.5.16, 2.5.17)
Source-to-detector distance	1183.4 mm	1500 mm
Source-to-isocentre distance	645 mm	1000 mm
Reconstruction diameter	700 mm	464.9 mm
Pixels	512 × 512	512 × 512
Resolution	1.287–1.367 mm	0.908 mm
Slice thickness	3 mm	2 mm
Kilovolt peak	120 kV	125 kV
Tube Current	196–310 mA	20 mA

### Dose evaluation procedure

**Fig. 1:**
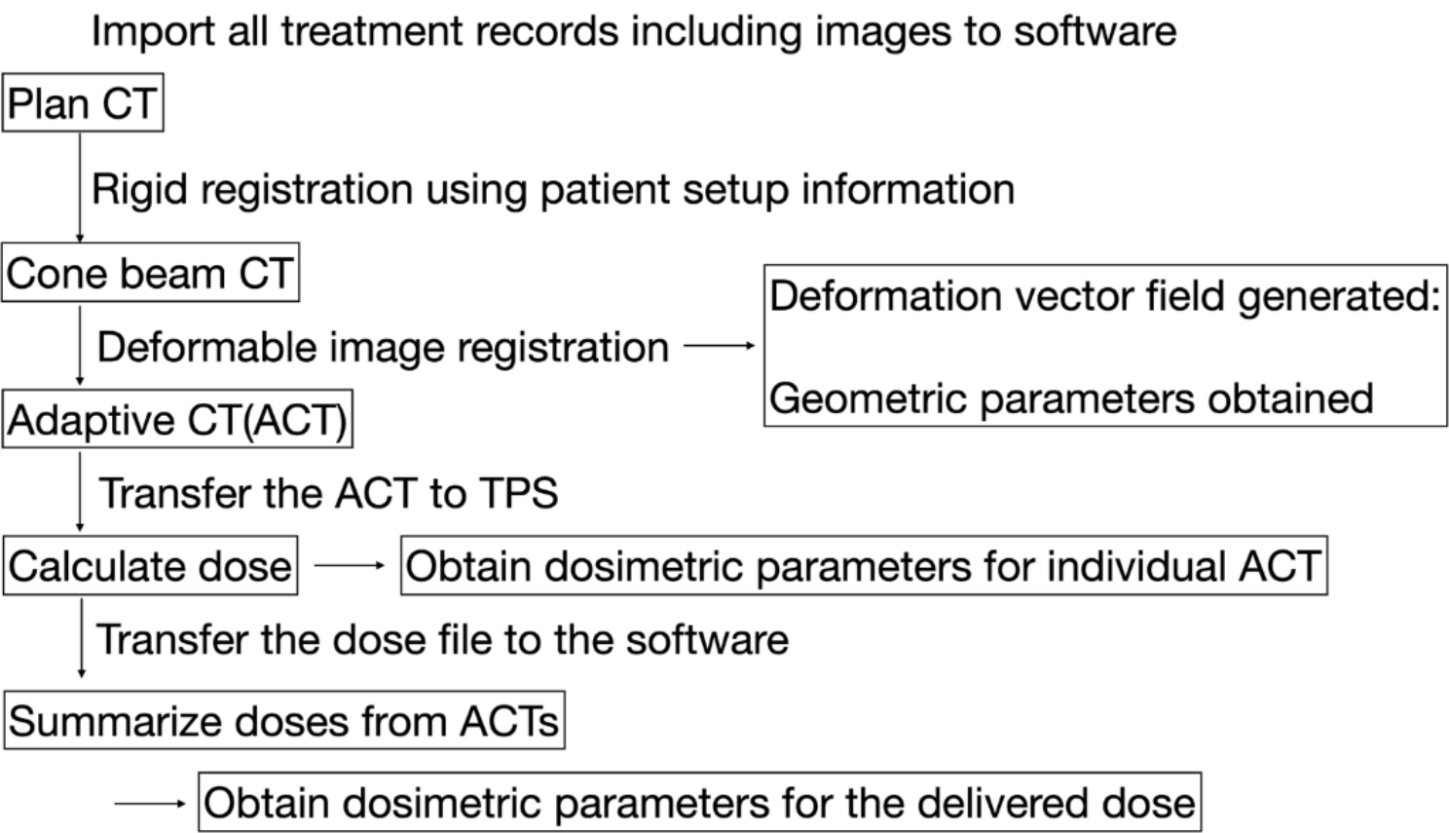
Workflow of the dose evaluation procedure

ACT generation began by aligning the PCT with CBCT. To estimate the dose delivered to the patient at the time of treatment, the couch location recorded by the CBCT matching to the PCT was used for rigid registration. The daily CBCT images were acquired with a free-breading. After applying rigid registration, deformable image registration of the PCT on CBCT was performed using the multipass B-spline algorithm [37, 38] in the Velocity software. The RS, including the ITV, PTV, and normal structures, was automatically propagated following the deformable vector field (DVF) of the PCT to the CBCT to generate adaptive RS (aRS) on the ACT. The ACT and aRS were then transferred to the TPS to calculate the delivered dose (aRD), reflecting the daily variations from the initial simulation. After the dose calculation was completed in the TPS, the accompanying aRD with the ACT was copied back to the Velocity software to deform the aRD back to the PCT coordinate. Each patient’s record contained four CBCTs, thus resulting in four ACTs and corresponding four aRDs. Therefore, the total dose delivered to the patient was estimated by averaging the four aRDs in the PCT coordinates. The workflow of the dose evaluation procedure is summarised in Fig. 1.

### Data analysis with parameters

Through the dose evaluation procedure described earlier, parameters related to the ITV and PTV were collected, as summarised in Table 2. These parameters were obtained from the dose statistics of the RD and aRDs in the TPS, deformable quality assurance [39], comparison of the RS and aRS, and from the dose accumulation processes using Velocity software. Dose-evaluation indices, such as homogeneity index [40], were calculated and used in the analysis. Obtained parameters were assessed to determine their relationship to the treatment target, specifically the dose delivered to the 95% volume of the PTV (D_95%_), using a stepwise multiple linear regression model in SPSS^®^ (Ver. 27, IBM^®^, Chicago, IL, USA). As the study assessed the treatment records of 72 patients, a total of 288 ACT cases was analysed. Through the analysis, significant parameters that passed the normality test and multicollinearity check were selected, and their p-values were reported. Additionally, analysis of variance (ANOVA) was performed to examine the mean difference between the parameters depending on the tumour location.

**Table 2:** List of parameters collected for internal and planning target volume

Source of Parameters	Internal target volume	Planning target volume
TPS	Equivalent sphere diameter, volume, planned dose (min, mean, max)	Equivalent sphere diameter, volume, planned dose (min, mean, max), D_2%_, D_95%_, D_98%,_ V_100%_, V_50%,_ lung lobe where the PTV is located
Deformable image registration	Delivered dose (min, mean, max), subtracted (delivered-planned) dose (min, mean, max)	Dice similarity coefficient (DSC = 2 × |PCT∩ACT| / (|PCT|+|ACT|), also called as conformality), surface distance (mean, standard deviation, max), Warp (min, mean, max), log Jacobian (min, Q1, median, Q3, max, MMR, IQR, delivered dose (min, mean, max, D_2%_, D_95%_, D_98%_), subtracted (delivered-planned) dose (min, mean, max)
Dose evaluation index		Homogeneity index (HI = (D_2%_ - D_98%_)*100/ D_prescribed_ [40]) and ΔHI = HI_delivered_-HI_planned_, conformity index (CI = V_100%_/volume), gradient index (GI = V_100%_/V_50%_), gradient measure (GM = (3×V_50%_/4π)^1/3^-(3×V_100%_/4π)^1/3^

*D_2%_, D_95%_, D_98%_: dose delivered to the 2, 95, and 98% volumes of the PTV; V_100%_, V_50%_: volume covering isodose level of 100% and 50% of the prescribed dose; MMR: max-min range; IQR: inter quartile range

### Statistics of the parameters

After the multiple linear regression analysis, the PTV parameters proven to be significant were examined. First, basic histograms of the PTV parameters including PTV D_95%_ and two-dimensional distributions of the parameters were presented. Volumes of the ITV and PTV were reviewed in the PCT and ACTs, and the average volume differences between the ACTs and PCT were investigated.

**Fig. 2:**
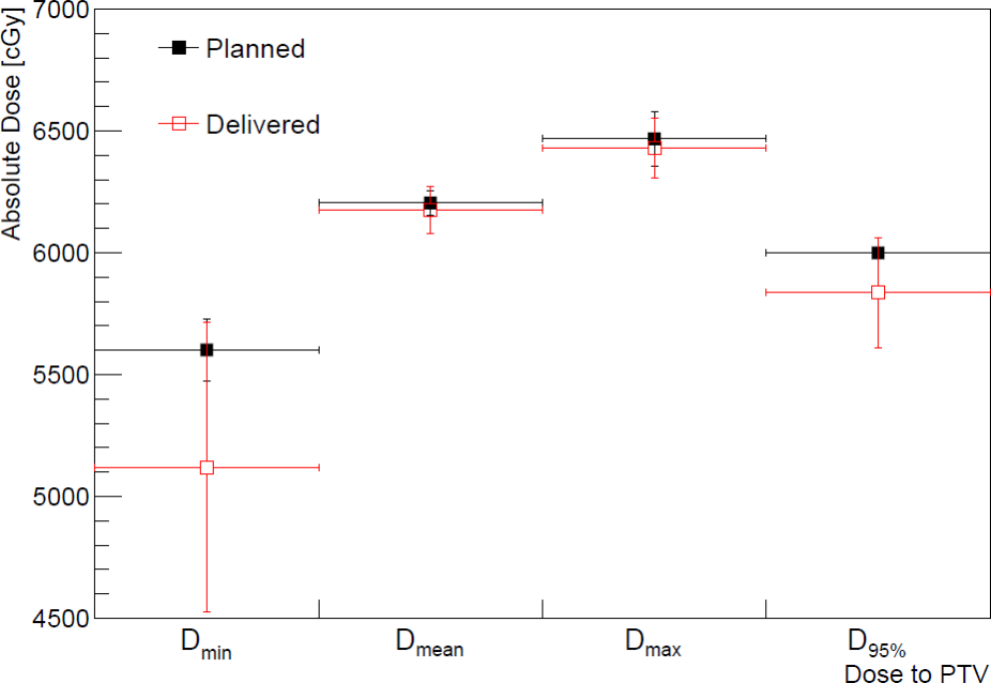
Dose comparison between planned (filled square) and estimated delivered (open square) dose to planning target volume (PTV) and their standard deviations: minimum (D_min_), mean (D_mean_), maximum dose (D_max_) to the PTV, and the dose delivered to the 95% volume of the PTV (D_95%_)

## Results

### Delivered dose evaluation

**Fig. 3:**
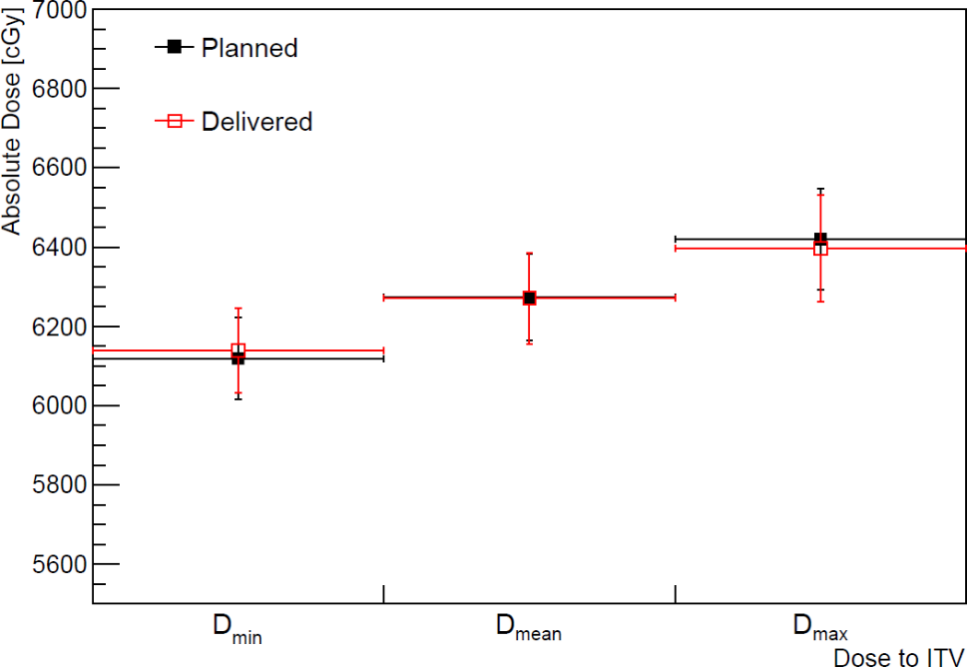
Dose comparison between planned (filled square) and estimated delivered (open square) dose to internal target volume (ITV) and their standard deviations: minimum (D_min_), mean (D_mean_), maximum (D_max_) dose delivered to the ITV

The dose delivered to the PTV and ITV was estimated from the aRDs and compared with the planned dose shown in Figs. 2 and 3, respectively. The planned and delivered doses were agreed within the uncertainty of the delivered doses. From a frequentist’s statistical perspective, the delivered dose to the PTV agreed with the planned dose within 6% at a 95% confidence level. Figure 3 shows a comparison of the delivered and planned doses to the ITV, and their maximum difference was found to be only 1%.

### Tumour volume change

**Fig. 4:**
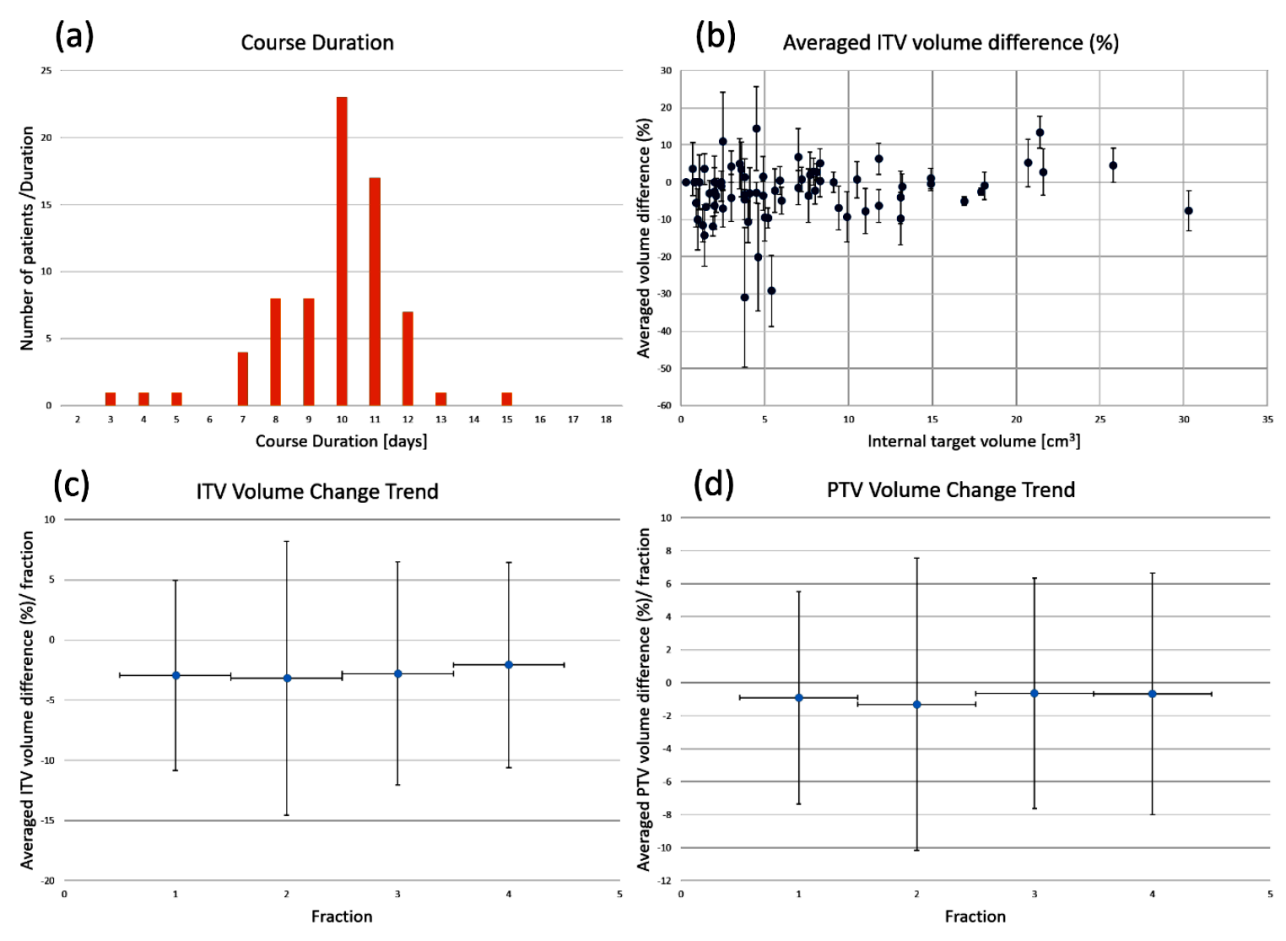
(**a**) Number of days taken for the stereotactic ablative radiotherapy of NSCLS patients, (**b**) scattered plot of their averaged difference and its standard deviation of the internal target volume (ITV, %) described in the adaptive CTs compared to the volume of the ITV in the treatment planning CT (PCT) depending on the ITV volume in the PCT, and their averaged volume difference and its standard deviation of the (**c**) ITV and (**d**) planning target volume (PTV) per fraction

Figure 4 shows the duration of SABR treatment for the 72 patients, the average volume difference of the ITV derived from the ACTs as a function of the ITV volume in the PCT, and the trends in the average volume differences between the ITV and PTV per fraction. Determining a definitive trend in the volumetric changes of the ITV and PTV proved challenging, as the observed standard deviation (SD) was substantial compared with the mean percentage difference.

### Parameters related to the treatment target

Four parameters were identified as significantly correlated with the dependent variable, PTV D_95%_, based on the multiple linear regression analysis. From the analysis, coefficients of the selected parameters are presented in Table 3 and were confirmed not to have multicollinearity. The parameters are the homogeneity index difference (**ΔHI)**, mean delivered dose to PTV (**D**_**mean**_), mean displacement of the deformed PTV voxels (**Warp**_**mean**_), and Dice similarity coefficient (**DSC**) between the PTV structures from PCT and ACT.

**Table 3:** Results of the multiple linear regression

Model	Unstandardised coefficients		Standardised coefficients	*P*-value	Tolerance	VIF
	B	Std. error	β			
Constant	2032.97	572.31		0.000		
ΔHI	-21.49	1.08	-0.648	0.000	0.513	1.948
D_mean_ [%]	33.45	5.30	0.191	0.000	0.601	1.664
DSC	558.55	149.40	0.111	0.000	0.623	1.605
Warp_mean_ [mm]	-33.71	9.78	-0.128	0.001	0.395	2.533
Adjusted R^2^	0.843					

*VIF: Variance Inflation Factor, Tolerance: a measure of collinearity, ΔHI: difference of homogeneity index, D_mean_: mean delivered dose to PTV, DSC: Dice Similarity Coefficient, Warp_mean_: mean displacement of PTV

**Fig. 5:**
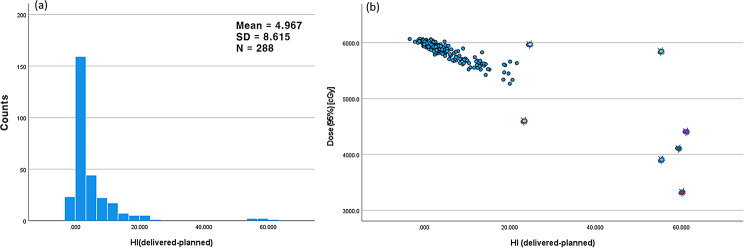
(**a**) One dimensional distribution of the homogeneity index difference (ΔHI) and (**b**) scattered plot of the delivered dose to 95% volume of the PTV (D_95%_) vs. ΔHI. Concerned outlier cases were highlighted in the scatter plot

**Fig. 6:**
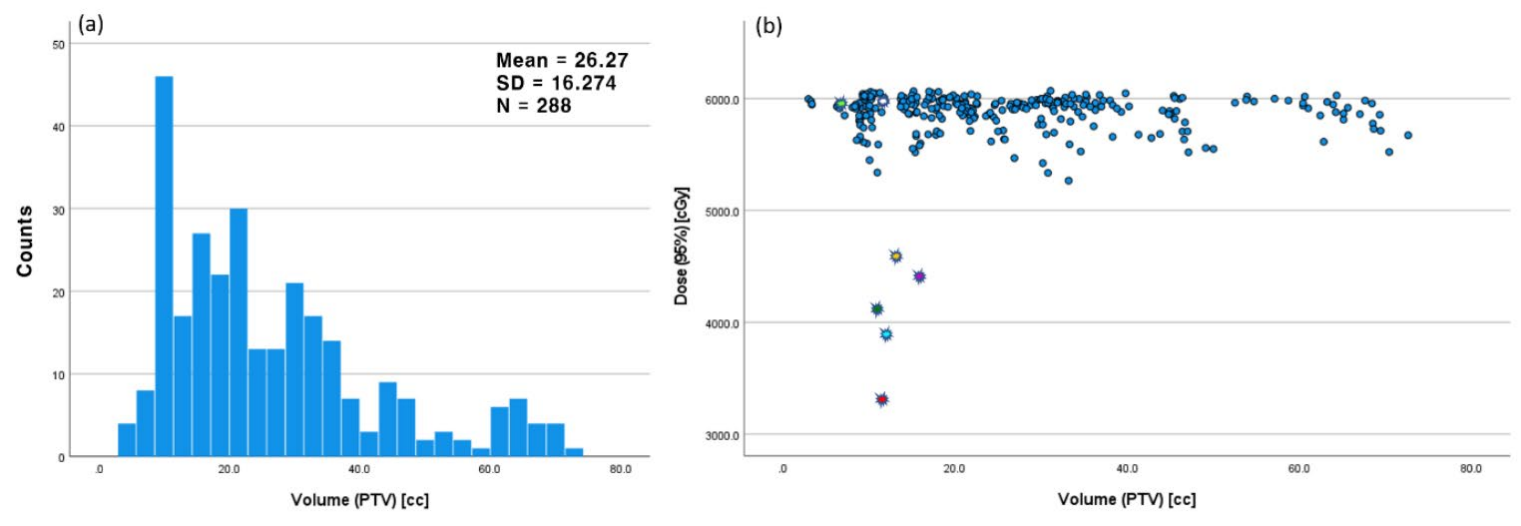
(**a**) One dimensional distribution of the PTV volume (cc) and (**b**) scattered plot of the delivered dose to 95% volume of the PTV (D_95%_) vs. the PTV volume. Concerned outlier cases were highlighted in the scatter plot

**Fig. 7:**
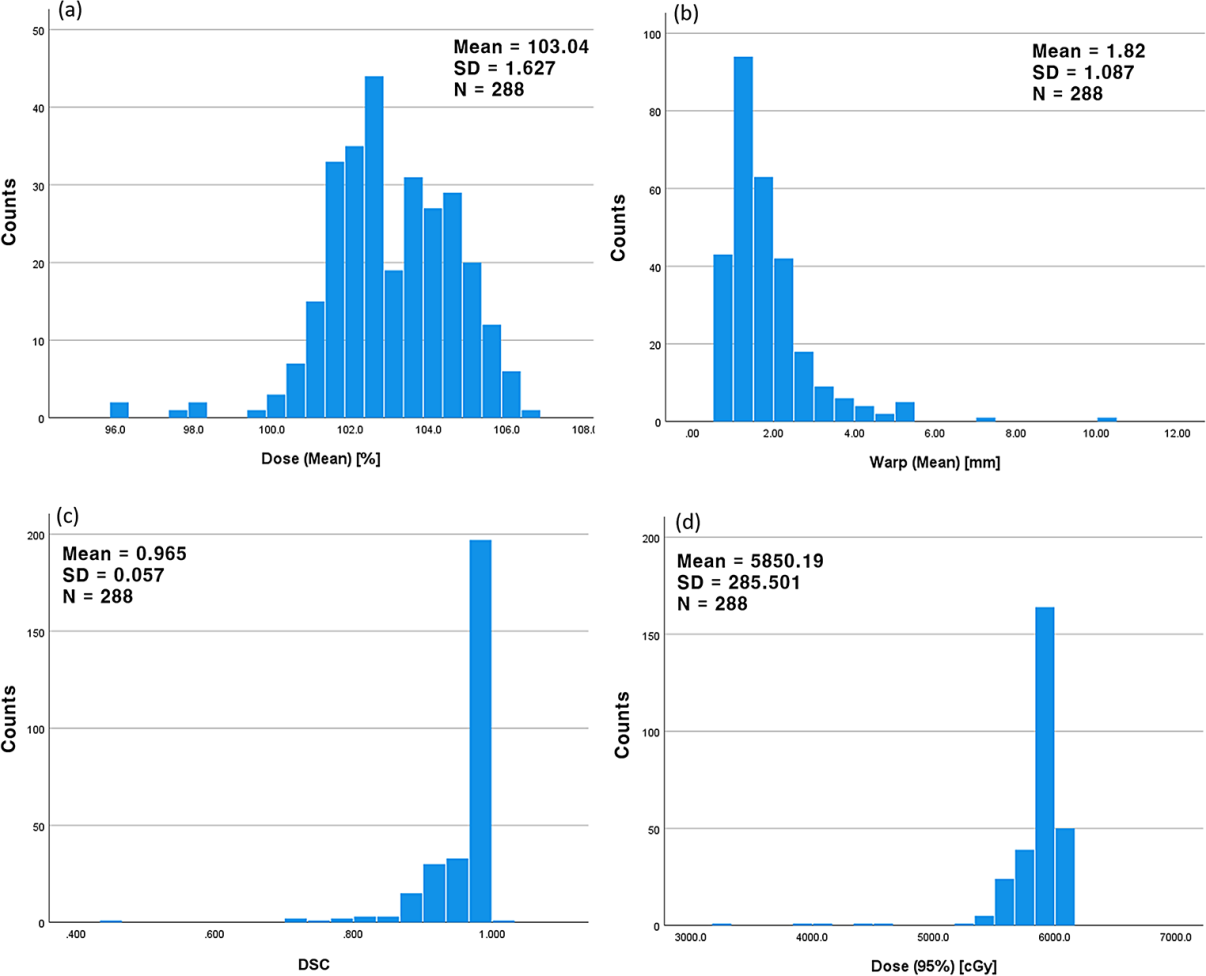
One-dimensional distribution of the independent parameters: (**a**) mean delivered dose to PTV (D_mean_), (**b**) mean Warping distance (Warp_mean_), (**c**) Dice coefficient of similarity (DSC), and a dependent parameter (**d**) delivered dose to 95% volume of the PTV (D_95%_)

Analysing all ACT cases, Figs. 5 and 6 demonstrate one-dimensional distributions of ΔHI and PTV volume and their relations with the PTV D_95%_ in scattered plots. Seven cases of the highest ΔHI, designated as outliers, are highlighted with sparkles in the plots. As shown in Fig. 6(b), these outliers have smaller PTV volumes. Figure 7 presents the one-dimensional distributions of the PTV D_mean_, Warp_mean_, DSC, and the dependent variable PTV D_95%_.

### Relations between the tumour location and PTV parameters

Each lung is divided into sections known as lobes that are closely associated with various structures within the thoracic cavity. The diaphragm, a dome-shaped primary respiratory muscle, is expected to undergo the most significant respiratory motion. Given that the diaphragm is located beneath the lower lung lobes, there has been specific interest in exploring the dependence of the values of PTV parameters, for example, the estimated delivered dose vs. tumour location. By assessing the mean values of the PTV parameters from 288 ACT cases, a significant difference was observed in PTV displacement and PTV D95% between the lobes (P-value less than 0.05) in Table 4.

**Table 4:** Means and standard deviations of the D_95%_, DSC, and Warp_mean_ distributions of the PTV depending on the tumour location in the lung lobe. Meaningful differences were assessed by the *P*-value

Lung Lobe	*N*	D_95%_±SD [cGy]	DSC ± SD	Warp_mean_±SD[mm]
LUL	84	5920.0 ± 147.6	0.97 ± 0.04	1.57 ± 0.83
LLL	60	5775.1 ± 439.4	0.96 ± 0.06	1.92 ± 1.22
RUL	64	5889.5 ± 120.9	0.97 ± 0.04	1.51 ± 0.68
RML	16	5806.5 ± 189.7	0.98 ± 0.04	2.47 ± 1.07
RLL	64	5800.5 ± 349.8	0.96 ± 0.08	2.20 ± 1.39
Total	288	5850.2 ± 285.5	0.97 ± 0.06	1.82 ± 1.09
*P*-Value		0.012	0.189	0

LUL, left upper lobe; LLL, left lower lobe; RUL, right upper lobe; RML, right middle lobe; RLL, right lower lobe

## Discussion

### Tumour volume change

Previous studies on NSCLC SABR treatment have reported changes in tumour volume, despite a short treatment duration of typically 7–12 days. Using various imaging modalities, some studies [41, 42] reported a consistent decrease in GTV, whereas others [43–45] noted a slight initial increase in GTV during the treatment course, followed by a decrease. At our institute, treatment records document the ITV instead of the GTV; therefore, this study investigated the changes in the ITV volume during the course of treatment. Considering the average volume differences per fraction for both the ITV and PTV are nearly zero and significantly smaller than the SD in Fig. 4(c) and (d), volume changes during the SABR treatment did not constitute a significant concern in this study.

In the ACTs, the ITVs and PTVs were automatically recontoured following the DVF obtained from the DIR using commercial software, and their volumetric differences were consistently negative. These plots indicate that the recontoured structures exhibit a marginally smaller volume than their original volume, and the volume difference may originate from the differences in the method of treating the respiratory motion between PCT and CBCT. The ITV in the PCT was delineated based on the MIP of the GTV, whereas the CBCT was acquired with free-breathing. This might have affected the ITV in the ACT, reflecting that the volumes recorded in the free-breathing CBCT are marginally smaller than the ITV and PTV in the PCT. However, this volume difference was significantly smaller than the SD. Comparing the values in Fig. 4(c) and (d), the average ITV differences are approximately twice as large as those of the PTV. These discrepancies might have occurred because the ITV, when used as the denominator to calculate the volume difference, is smaller than the PTV.

### Recontoured structures for dose evaluation

Concerns may arise regarding the accuracy of the aRS recontoured automatically using the commercial software. When applying the ACT for adaptive planning, it is essential for a radiation oncologist to review and correct the delineation of the ITV and PTV prior to treatment approval. Nonetheless, the automatically recontoured ITV and PTV on ACT, based on the DVF, were considered adequate for evaluating the dose delivered to the patients. This study was performed to investigate how the original PTV was deformed in the treatment and whether the planned dose was actually delivered to the original PTV. Among the collected patient data, the ITV and PTV delineations were randomly selected and reviewed by an expert for comparison with manual recontouring. This comparison revealed no significant differences in dosimetric evaluation, thus supporting the use of automatically recontoured structures for dose evaluation across all 288 ACT cases.

### PTV parameters depending on the tumour location

The PTV located in the lower lobes showed a greater discrepancy in the position between the treatment and simulation. The mean Warp_mean_ value was notably higher and the mean D_95%_ value was significantly lower for tumours located in the lower lobes compared with those of tumours in the upper lobes (Table 4). Furthermore, the SDs for PTVs in the lower lobes exhibited a significant increase, indicating that tumours located in the upper lobes allow for more consistent patient setup reproducibility, thereby enhancing the accuracy of the prescribed dose delivery to the PTV. Among the seven outliers, six had PTV located in the lower lobes. This may be attributed to the observed higher Warp_mean_ and lower D_95%_ values in the lower lobes, a trend that these outliers similarly exhibited.

### Outliers of the multiple linear regression model prediction

**Fig. 8:**
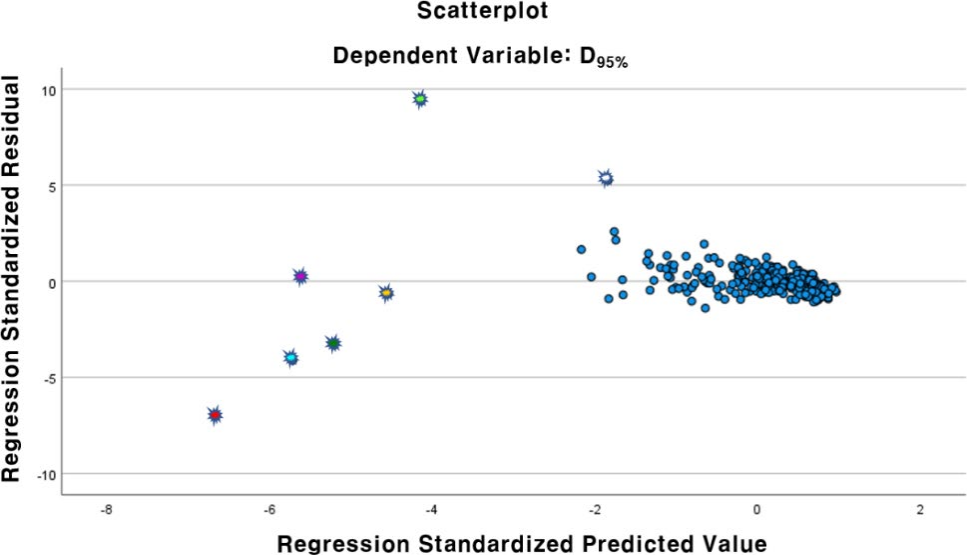
Scattered plot of the residual of the dose prediction vs. the predicted valued of the regression model. Concerned outliers were highlighted in the scatter plot

Seven outliers marked in Figs. 5 and 6 are characterised by having the highest ΔHI and small PTV volume. These outliers show significant deviations from linearity, as shown in Fig. 8. The PTV D_95%_ values of the outliers calculated from the ACTs in the TPS deviated from the PTV D_95%_ predictions of the multiple linear regression. Two geometric and two dosimetric parameters were found to be significantly related to the D_95%_ in the multiple linear regression, and the detailed results are listed in the Table 3.

To evaluate the effect of ACTs with a high ΔHI, stepwise multiple linear regression was repeated, excluding ACT cases of HI > 17, based on the distribution in Fig. 5(b). The results summarised in Table 5 indicate that only the dosimetric parameters maintained a significant correlation with D_95%_. This indicates that the outliers may be closely linked to the two geometric parameters that represent the PTV displacement. Such displacement might imply uncertainties from the patient setup and respiratory motion, resulting in dosimetric uncertainty. Kim et al. [46] highlighted that geometric uncertainties in patient positioning can limit the clinical advantages of IMRT. Therefore, during SABR treatment, a more thorough consideration of patient setup and respiratory motion is imperative.

Although these cases exhibited suboptimal dosimetric outcomes for the PTV, Fig. 3 confirms that the dose was accurately delivered to the ITV. This demonstrates that the margin between the ITV and PTV effectively ensures sufficient dose coverage of the ITV. As the patients were treated under the prescription of one radiation oncologist, the ITV and PTV were consistently determined. However, it is worth studying whether the margin can be further reduced with respect to various parameters obtained. In this study, analysis of multiple parameters enabled a more precise evaluation of the dose delivered.

**Table 5:** Results of the multiple linear regression by SPSS^®^ (v27, IBM^®^, Chicago, IL, USA) with cases having ΔHI less than 17

Model	Unstandardised coefficients		Standardised coefficients	*P*-value	Tolerance	VIF
	B	Std. error	β			
Constant	3924.36	240.32		0.000		
ΔHI	-26.98	0.92	-0.790	0.000	0.771	1.297
D_mean_ [%]	19.98	2.31	0.232	0.000	0.771	1.297
Adjusted R^2^	0.85					

*VIF: Variance Inflation Factor, Tolerance: a measure of collinearity, ΔHI: difference of homogeneity index, D_mean_: mean delivered dose to PTV

### Uncertainties affecting the deformed image

In DIR, the PCT is a moving image, while the daily CBCT is a stationary image. When the PCT is deformed to the daily CBCT, the deformed PCT is called an ACT, retaining patient information at the time of the treatment setup. In the TPS dose calculation, the delivered dose based on the ACT was calculated using the original beam plan. Thus, we believe that the setup error implied by the ACT affects the estimated delivered dose.

Although the QA parameters of DIR [39] were assessed and included in the analysis (Table 2), the uncertainty of the DIR algorithm was not considered. Repeating the DIR using the same CBCT and PCT images is insufficient to estimate the DIR uncertainty; however, the DIR uncertainty is expected to be systematic without giving a rise to an outlier. As the outliers of interest occurred randomly among the 288 ACT cases, they likely originated from a random source, such as the setup error rather than the DIR algorithm error.

### Limitations of this study

One limitation is the resolution of the parameters obtained by comparing the images. The image resolutions of the PCT and daily CBCT are presented in Table 1. Recalling the method of generating the ACT, it is a result of the deformation of the PCT to the daily CBCT, thus the resolution of the ACT is same as that of the PCT; the transverse and vertical resolutions of the ACT were approximately 1.3 and 3 mm, respectively. Considering the Warp_mean_, which showed a peak around 1.3 mm (Fig. 7b), it is challenging to calculate any finer displacement. However, we used the average value of the displacement calculated from PTV voxels; thus, we did not rely on a single movement value but on the movement trend of the PTV structure.

Secondly, a methodology covering intra-fractional motion during SABR was lacking. Monitoring the intra-fractional motion might be optimal for IMRT treatment using an MR-Linac. In the CBCT-Linac option, it is difficult to track real-time respiratory motion during treatment. In particular, for SABR, respiratory motion-controlled treatments, such as DIBH, are not normally considered. Although we observed one case of using continuous positive airway pressure breathing, the respiratory motion was not monitored.

Intra-fractional motion was considered as the respiratory motion in the PCT and ACT images, and the patients were subjected to treatment with a free-breathing. In PCT, the respiratory motion was assessed using the MIP of the tumour. As we did not control the patient’s respiration during CBCT acquisition, which took approximately 1 min, the image was expected to represent the average motion of the GTV. Surrounding OARs were also obtained with average intensity projection (AIP) on PCT and with the free-breathing on CBCT.

### Comparison of the result with previous studies

Previous studies utilised the same commercial software to evaluate the dose delivered to patients with NSCLC. Czajkowski P et al. [35] evaluated the accuracy of dose delivery in the stereotactic radiation therapy for both brain and lung cancers. Although they analysed only 10 patients for the lung SABR dose evaluation, they reported no significant change on the ITV volume during the treatment and agreement within ± 10% on the dose in 99% volume of the PTV between the PCT and ACT. They assessed only the PTV volume and DSC regarding the PTV change. On the contrary, Wang B et al. [36] investigated the differences of delivered and planned dose to PTV as well as to the OARs, which were clinically acceptable. They used records of 27 patients with locally advanced NSCLC who were treated with 51 Gy in 17 fractions. They generated ACTs for treatment in 1,5,9,13, and 17. A significant tumour shrinkage (11.1%) was observed through the course. However, no significant difference was discovered in the volume of 51 Gy isodose line corresponding to the PTV, but limited increase (< 5%) was observed in total lung, oesophagus, and heart.

Compared with previous studies, we calculated delivered dose for every fraction and assessed the PTV parameters as much as possible with an increased number of patients. We were able to obtain distributions of the significant PTV parameters and the parameters were proven to be related to the PTV D_95%_. Besides the delivered dose evaluation, the PTV D_95%_ and the PTV displacement from the PCT were observed significantly related to the tumour location.

## Conclusions

Throughout the evaluation of the delivered dose, we confirmed that the prescribed dose was successfully delivered to the ITV. The analysis showed that the HI difference between the ACT and PCT was the most sensitive parameter for the delivered PTV D_95%_. Although the dose was delivered to the PTV successfully in most cases, a few outliers with higher ΔHI, that degraded the PTV dose distribution, were observed. Judging by the relationship between geometric parameters of the PTV and the worse PTV D_95%_, these outliers might be caused by the misaligned patient setup. Analysis of the delivered dose along with the parameters obtained during the evaluation process demonstrated that the PTV margin effectively compensated for the setup uncertainty.

## Data Availability

No datasets were generated or analysed during the current study.

## Abbreviations

ACT: Adaptive CT, so called as synthetic CT
ANOVA: Analysis of variance
CT: Computed tomography
CBCT: Cone beam CT
D2%/ D95%/ D98%: Dose delivered to 2 %, 95 %,/ 98 % of the PTV
Dmin/ Dmean/ Dmax: Dice similarity coefficient
DVH: Dose volume histogram
4DCT: Four dimensional CT
GTV: Gross tumour volume
HI: Homogeneity index
IGRT: Image-guided radiation therapy
IMRT: Intensity-modulated radiation therapy
ITV: Internal target volume
MIP (AIP): Maximum (average) intensity projection
NSCLC: Non-small-cell lung cancer
OAR: Organ at risk
PTV: Planning target volume
PCT: Planning CT or reference CT
RP, RD, RS: Dicom RT plan (RP), RT dose (RD), and RT structure (RS) accompanied by PCT
aRD, aRS: Dicom RT dose (aRD) and RT structure (aRS) accompanied by ACT
SABR(SBRT): Stereotactic ablative radiotherapy (stereotactic body radiation therapy)
SD: Standard deviation
TPS: Treatment planning system
War_pmean_: Mean displacement of the PTV voxels
